# Green synthesis of zinc oxide nanoparticles using *Rhus coriaria* extract and their anticancer activity against triple-negative breast cancer cells

**DOI:** 10.1038/s41598-024-63258-7

**Published:** 2024-06-12

**Authors:** Youssef Mongy, Thanaa Shalaby

**Affiliations:** 1 Department of Applied Medical Chemistry, Medical Research Institute, Alexandria University, Alexandria, 21561 Egypt; 2 Medical Biophysics Department, Medical Research Institute, Alexandria University, Alexandria, 21561 Egypt; 3 Nanotechnology Training Center, Medical Technology Center, Alexandria University, Alexandria, Egypt

**Keywords:** Zinc oxide, Nanoparticles, Green synthesis, *Rhus coriaria L*., Anticancer activity, Breast cancer, Breast cancer, Nanoparticles

## Abstract

The growing interest in using plant extracts for the biogenic synthesis of zinc oxide nanoparticles (ZnO NPs) stems from their facile, eco-friendly, and biologically safe approach instead of chemical routes. For the first time, ZnO NPs were successfully biosynthesized using *Rhus coriaria* fruit aqueous extract as a reducing and capping agent. Characterization revealed that the biosynthesized ZnO NPs possessed a maximum absorbance of approximately 359 nm and closely resembled the hexagonal ZnO wurtzite crystalline structure, with an average crystalline size of 16.69 nm. The transmission electron microscope (TEM) showed the presence of spherical and hexagonal morphologies, with an average grain size of 20.51 ± 3.90 nm. Moreover, the elemental composition of the synthesized ZnO NPs was assessed via energy-dispersive X-ray spectrometry (EDX), and the presence of phytocompounds on their surface was subsequently verified through FT-IR analysis. The ζ-potential of ZnO NPs was recorded at − 19.9 ± 0.1663 mV. Regarding anti-cancer properties, ZnO NPs were found to possess potent anti-tumor effects on MCF-7 and MDA-MB-231 breast cancer cells. Their efficacy was dose-dependent, with IC_50_ values ranging from 35.04–44.86 μg/mL for MCF-7 and 55.54–63.71 µg/mL for MDA-MB-231 cells. Mechanistic studies in MDA-MB-231 cells revealed apoptosis induction, validated by DAPI staining, confocal microscopy, and Annexin V/PI staining, showing apoptosis by 12.59% and 81.57% at ½ IC_50_ and IC_50_ values, respectively. Additionally, ZnO NPs were observed to provoke S-phase arrest and inhibit colony-forming and metastatic potential by modulating apoptosis and metastasis-related genes. This study unravels new insights into how ZnO NPs provoke cancer cell death and inhibit metastasis, revealing new prospects in cancer nanotechnology.

## Introduction

Nanotechnology has gained noteworthy consideration since nanoscale materials exhibit distinctive chemical and physical properties, surpassing bulk materials^1^. For this purpose, nanoparticles (NPs) can be integrated into multiple applications in diverse fields, including medicine^2^. Among different types of NPs, metal oxide NPs play an integral role in biomedical areas, including drug delivery, imaging, biosensors, and other applications^3,4^. Within the metal oxide NPs, zinc oxide NPs (ZnO NPs) generally engage considerable interest owing to their unique physicochemical characteristics, such as their high surface area, high exciton binding energy, and photocatalytic activity^5^. They have versatile applications across various fields, such as energy preservation, textile production, electronics, catalysis, cosmetics, and semiconductors^6,7^. Moreover, they have diverse biomedical applications, including antimicrobials, drug delivery, wound healing, and biosensing^6,7^.

Breast cancer is a heterogeneous malignancy and is considered the most widespread cancer in women, with a high mortality rate^8^. It can be categorized into distinct molecular subtypes, according to the expression levels of estrogen, progesterone, and human epidermal growth factor receptor 2 receptors^9^. Due to the adverse effects associated with conventional chemotherapy, considerable attention has been directed towards developing novel therapeutic strategies for treatment with improved side effects. In recent decades, nanotherapy has emerged as one of the promising modalities^10,11^. In the early research stages, NPs served as carriers for anti-cancer drugs, wherein anti-cancer medications were encapsulated. Following this, the intrinsic anti-tumor potential of NPs was revealed, prompting their direct application as therapeutic agents against tumor growth. One of the promising intrinsic anti-tumor agents is ZnO NPs as multiple studies have been published demonstrating their cytotoxicity against tumor cells^12,13^. They can induce selective inhibition of cancer cells with additive or synergistic effects with anti-cancer compounds^14,15^.

Despite the various chemical methods employed to produce ZnO NPs, most of these approaches pose unfavorable aspects to the environment as harsh, dangerous, and toxic chemicals are used, besides the high cost and complicated reaction conditions^16,17^. In response, green synthesis of ZnO NPs has attracted researchers’ attention over chemical methods owing to its unique characteristics as it is environmentally friendly, facile, and devoid of expensive, harsh, and toxic chemicals^18^. One of the major green approaches is using plant extracts, a substantial tool as an effective and non-toxic technique for the facile green synthesis of ZnO NPs.

*Rhus coriaria* L. (*R. coriaria*) fruit is a member of the *Anacardiaceae* family, commonly known as sumac, and has been widely consumed as a traditional condiment and herbal medicine for decades in the Mediterranean region^19^. *R. coriaria* has gained increased interest in recent years due to its therapeutic outcomes. Owing to the existence of phytochemical compounds, such as phenolic acids, flavonoids, and tannins^20,21^, many health benefits are attributed to sumac, such as anticancer, antioxidant, antimicrobial, anti-inflammatory, antifungal, hypoglycemic, hypolipidemic, neuroprotective, and anti-atherogenic properties^21,22^.

To the best of our knowledge, no study has been published yet on the eco-friendly synthesis of ZnO NPs using *R. coriaria* fruit extract. Herein, we report a novel, facile, and cost-effective approach for the biosynthesis of ZnO NPs using *R. coriaria* fruit aqueous extract. The optical, structural, morphological, compositional, functional, and surface charge characteristics of the formed NPs were comprehensively studied. Furthermore, the antitumor effects of the obtained NPs were evaluated on MCF-7 and MDA-MB-231 breast cancer cells. Additionally, the mechanism underlying cancer cell death and metastatic potential induced by ZnO NPs was thoroughly investigated, which will provide novel opportunities in cancer nanotechnology.

## Results and discussion

### Biosynthesis and physicochemical characterization of ZnO NPs

The *R. coriaria* fruit extract represents an excellent and eco-friendly choice for biosynthesizing ZnO NPs. It displays a dual role by acting as a reducing agent and stabilizer, enhancing the successful synthesis of the NPs. At first, the formation of ZnO NPs was confirmed through visual observation of the solution color change to yellowish gray, followed by off-white. After that, the optical properties and other characteristics were extensively investigated through the subsequent analyses:

### Optical properties

**Figure 1:**
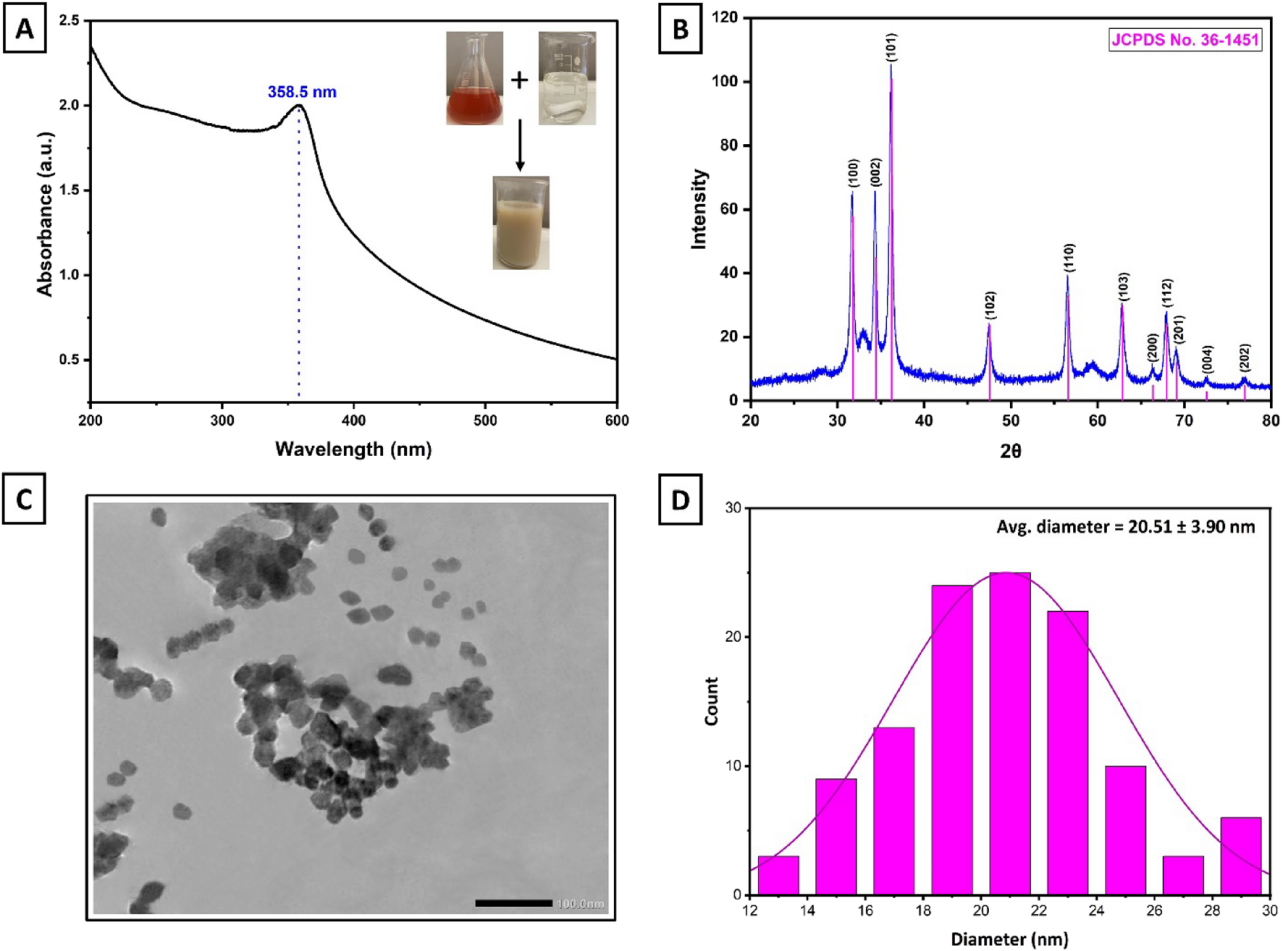
(**A**) UV–Vis spectral analysis of ZnO NPs in the 200–600 nm wavelength range. (**B**) XRD spectrum of ZnO NPs in the diffraction angle range of 20°–80°. (**C**) TEM image displaying the size and morphology of ZnO NPs. (**D**) The particle size distribution graph shows the average particle size of ZnO NPs.

Initially, UV–Vis spectroscopy was conducted to validate the biosynthesis of ZnO NPs in the wavelength region 200–600 nm at room temperature. As shown in Fig. 1A, the UV–Vis absorption spectrum revealed that the maximum absorbance existed at approximately 359 nm, representing the distinctive peak absorbance value for ZnO NPs. The surface plasmon resonance (SPR) of ZnO NPs was the cause of this peak, where the absorption of incident light is a result of the collective oscillation of free conduction band electrons^23^. Additionally, no additional peaks were observed in the spectrum, confirming the successful formation of pure ZnO NPs. The existence of an absorbance peak at 359 nm aligns with prior reports that ZnO NPs display optical absorption between 340 and 380 nm^23–25^.

### Structural properties

The structural formation, crystallinity, and crystallite size of synthesized NPs were also investigated by X-ray diffraction (XRD). As depicted in Fig. 1B, the XRD pattern displayed diffraction peaks of the green synthesized ZnO NPs and their Joint Committee on Powder Diffraction Standards (JCPDS). To start with, the diffractogram showed three intense diffraction peaks with 2θ values of 31.68°, 34.34°, and 36.17°, which are attributed to (100), (002), and (101) crystallographic reflection planes. Moreover, other strong diffraction peaks were also observed, showing 2θ values of 47.45°, 56.53°, 62.81°, 66.32°, 67.92°, 69.03°, 72.56°, and 76.93°, which correspond to (102), (110), (103), (200), (112), (201), (004), and (202) crystallographic reflection planes, respectively. These diffraction planes are in agreement with the indicated values of the hexagonal ZnO wurtzite crystalline structure (JCPDS No. 36–1451)^26,27^. Conversely, the diffractogram showed background humps at 2θ, which are frequently observed in green synthesis methods and can be linked to the surface crystallization of organic constituents from the fruit extract on the surface of NPs^28,29^. Furthermore, the Debye–Scherrer equation indicated that the biosynthesized ZnO NPs possessed an average crystalline size of 16.69 nm and a crystallinity percentage of 89.06%.

### Morphological properties

The transmission electron microscopy (TEM) analysis was performed not only to validate the findings from XRD mentioned earlier but also to determine the morphology and average grain size of the ZnO NPs produced through the eco-friendly approach. Figure 1C represents the TEM micrograph of the obtained NPs at a magnification of 100 nm while Fig. 1D shows the histogram of their average particle grain distribution size. As a result, ZnO NPs possessed spherical and hexagonal morphologies with mild aggregations, which aligns with prior reports^30,31^. Some NPs exhibited transparency while others appeared thick due to the NPs' overlap. The micrograph also displayed that the NPs were embedded within an active organic compound template sourced from the fruit extract, signifying their potential role in both the reduction and capping processes during the synthesis of ZnO NPs. In addition, the histogram reveals that the grain particle size of the NPs was in the range of 12–30 nm with an average of 20.51 ± 3.90 nm. This outcome matches the previously calculated crystalline size using Scherer's formula, as verified by the XRD result.

### Compositional properties

**Figure 2:**
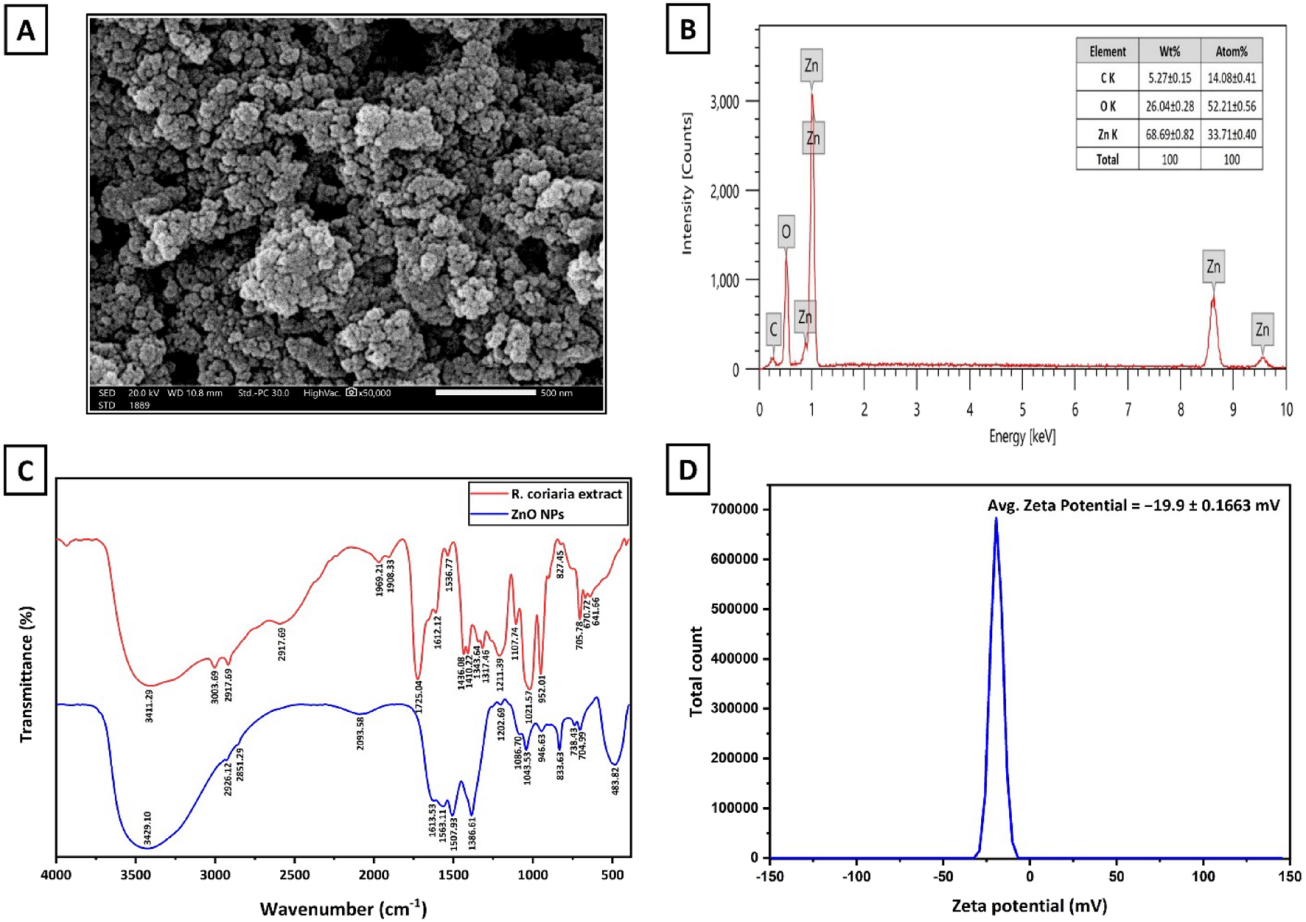
(**A** and **B**) SEM micrograph and EDX spectrum of ZnO NPs. (**C**) FT-IR patterns of *R. coriaria* fruit aqueous extract and ZnO NPs in the wavenumber range of 4000–500 cm^−1^. (**D**) Average zeta potential of ZnO NPs.

The elemental composition of the formed ZnO NPs was carried out using energy-dispersive X-ray spectrometry (EDX) along with scanning electron microscopy (SEM) (Fig. 2A and B). EDX spectra proved that the obtained ZnO NPs were mainly composed of Zn and O elements. Zinc exhibited three distinct peaks at approximately 1 keV, 8.7 keV, and 9.6 keV, while oxygen presented a single peak near 0.5 keV. These peaks are consistent with the characteristic signatures of ZnO NPs^29^. According to the elemental analysis, the weight percentages of Zn and O were 68.7% and 26%, respectively. In Figure 2B, the corresponding atomic percentages for Zn and O were found to be 33.7% and 52.2%, respectively. Additionally, a low-intensity signal, associated with carbon, was detected at 0.27 keV. This peak can be attributed to the X-ray emissions associated with the remaining organic phytochemical residues from the fruit extract^25,29^.

### Functional properties

Consequently, the existence of phytocompounds on the surface of ZnO NPs was further confirmed by Fourier Transform Infrared Spectroscopy (FT-IR) analysis. As presented in Fig. 2C, the FT-IR spectrum of *R. coriaria* fruit extract indicated the presence of characteristic bands indicative of the functional groups of its phytochemicals. Besides, the distinctive bands of biosynthesized ZnO NPs were analyzed between 4000 and 450 cm^−1^. Here, the resultant FT-IR spectrum showed a prominent band at 3429 cm^−1^, which correlates to the stretching vibration of the OH group of phenolic compounds^31^. Then, two small peaks were noticed at 2926 cm^−1^ and 2851 cm^−1^, corresponding to asymmetrical and symmetrical C–H stretching vibrations of the CH_2_ groups, respectively^32,33^. A singular band was observed at 2093 cm^−1^ representing the aromatic overtones, followed by a peak around 1613 cm^−1^, which is attributed to the C=C stretching vibration of conjugated alkene groups^34,35^. After that, two bands appeared at 1563 cm^−1^ and 1507 cm^−1^, corresponding to the C=C stretching vibration of aromatic compounds^34,35^. The C–H bending vibration was noted at a peak of 1386 cm^−1^. In addition, the presence of a peak at 1203 cm^−1^ can be indicative of the existence of C–O stretching vibrations associated with phenolic compounds, whereas two peaks at 1086 cm^−1^ and 1043 cm^−1^ are associated with the C–O–C stretching vibrations of polysaccharides and phenolic compounds^31,34^. Subsequently, the peak at 946 cm^−1^ represented the C–H vibrational bending of possibly the alkene group. Next, three peaks were observed at 833 cm^−1^, 738 cm^−1^, and 705 cm^−1^, indicating C–H bending vibrations, which may be associated with aromatic compounds^25^. Finally, the characteristic stretching vibration mode of Zn–O was obtained at 483 cm^−1^. Therefore, findings from the FT-IR analysis validate the presence of diverse active functional groups of phytochemicals on the surface of ZnO NPs.

### Surface charge properties

The zeta potential (ζ-potential) of biosynthesized NPs was measured. In general, ζ-potential provides a precise measurement of the net electrical charge of NPs present in the solution, reflecting the prospective long-term stability of the colloidal solutions^36^. It examines the dispersion or aggregation of particles, with a decrease in the ζ-potential values resulting in a decline in repulsive forces between the NPs, leading to the formation of large aggregates. According to Fig. 2D, the ζ-potential analysis revealed that ZnO NPs exhibited a negative charge at − 19.9 ± 0.1663 mV. Significantly, the observed ζ-potential value of the as-prepared NPs in this study surpasses the findings reported in other relevant studies^32,37,38^. It indicates the relative good stability and dispersity of the green synthesized ZnO NPs, which can be attributed to the presence of functional groups from *R. coriaria* fruit extract on their surfaces, serving the function of capping and maintaining the stability of NPs.

**Figure 3:**
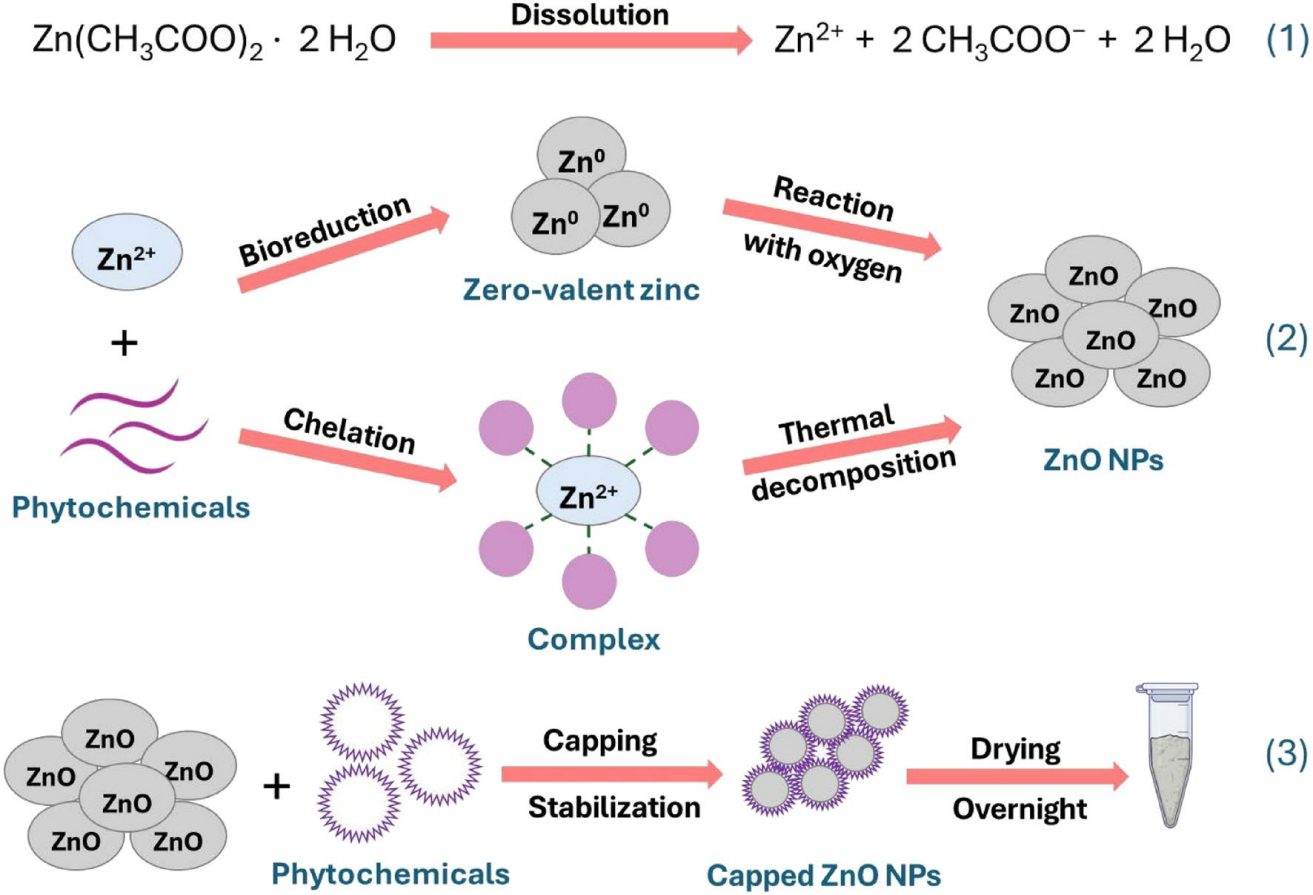
Proposed reaction mechanism of green synthesis of ZnO NPs using *R. coriaria* fruit aqueous extract.

*R. coriaria* fruit aqueous extract is an excellent, environmentally safe medium with versatile properties that enable it to function as a solvent, reducing agent, and stabilizer, facilitating the synthesis process of ZnO NPs. It has been reported that several bioactive components are present in the *R. coriaria* fruit extract, including hydrolyzable tannins, phenolic acids, anthocyanins, and flavonoids^39,40^. Within these compounds, gallic acid was found to be the most prevalent bioactive compound, succeeded by cyanidin-3-glucoside, the glycosides isoquercitrin, quercitrin, hyperoside, and ellagic acid^39^. The mechanism of biosynthesis of ZnO NPs using *R. coriaria* fruit aqueous extract remains unclear. In the literature, two synthesis mechanisms have been proposed for the eco-friendly synthesis of ZnO NPs using plant extracts^41,42^. The schematic reaction mechanism for the green synthesis of ZnO NPs is presented in Fig. 3. Initially, the dissolution of zinc acetate in water results in the liberation of freely moving ions. One mechanism involves the interaction between phytochemicals found in the extract and zinc ions, resulting in the formation of metal-coordinated complexes. These complexes undergo thermal treatment to break them down, leading to the formation of ZnO NPs. Alternatively, another proposed mechanism involves the bioreduction of Zn^2+^ ions by phytochemicals to metallic zinc, followed by a subsequent reaction with dissolved oxygen in the precursor solution, resulting in the formation of ZnO nuclei^43,44^. Additionally, the phytochemicals derived from the extract could serve as a capping and stabilizing agent, facilitating the stabilization of ZnO NPs.

### Anticancer activity of *R. coriaria* fruit aqueous extract and as-prepared ZnO NPs

**Figure 4:**
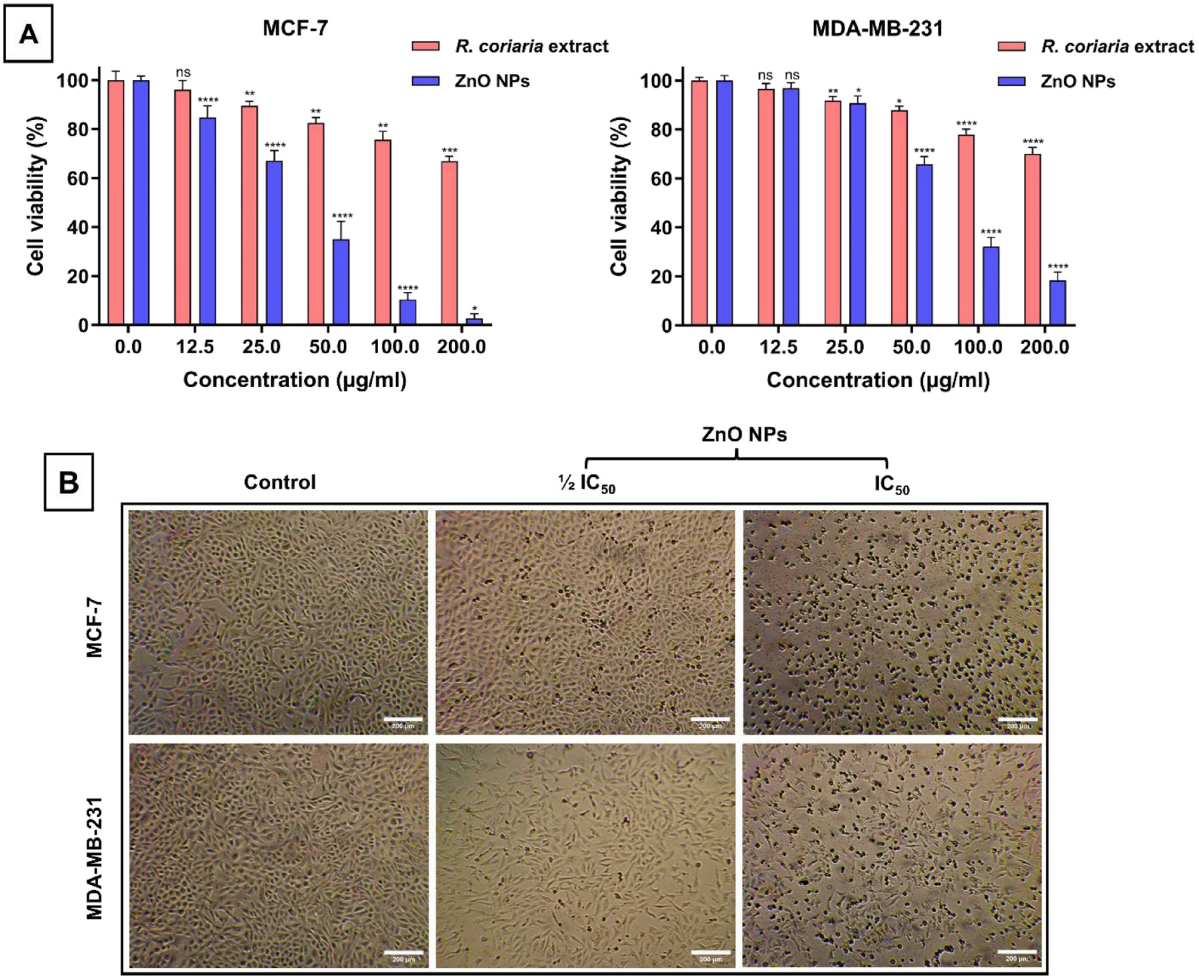
Anticancer activity of *R. coriaria* fruit aqueous extract and as-prepared ZnO NPs. (**A**) The cytotoxic effects of *R. coriaria* fruit aqueous extract and ZnO NPs on MCF-7 and MDA-MB-231, respectively. All data were presented as mean ± SD and analyzed using one-way ANOVA (Turkey’s test); ****P-value signifies < 0.0001, ***P-value signifies < 0.001, **P-value signifies < 0.01, and *P-value signifies < 0.05. (**B**) Microscopic images showing morphological alterations of MCF-7 and MDA-MB-231 cells after 24-h treatment with ½ IC_50_ and IC_50_ values of ZnO NPs.

In the present study, the cytotoxic effects of the green synthesized ZnO NPs and *R. coriaria* fruit aqueous extract were evaluated using two human breast cancer cell lines, MCF-7 and MDA-MB-231. The cells were treated with ZnO NPs and *R. coriaria* fruit aqueous extract at varying concentrations, ranging from 200 to 12.5 μg/mL, for 24 h. Then, cell viability percentages were determined through the 3-(4,5-dimethylthiazol-2-yl)-2,5-diphenyltetrazolium bromide (MTT) assay relative to the control groups. As shown in Fig. 4A, ZnO NPs possessed dose-dependent cytotoxic effects on both MCF-7 and MDA-MB-231 cells, where the growth of cancer cells experienced a marked decrease as the concentration of NPs increased. Remarkably, at lower concentrations of ZnO NPs, MCF-7 cells demonstrated significantly greater sensitivity and toxicity compared to MDA-MB-231 cells. However, upon exposure to a concentration of 200 μg/mL, the maximum anticancer activities of ZnO NPs were recorded, resulting in a cell viability percentage of 2.7% for MCF-7 cells and approximately 18.3% for MDA-MB-231 cells. Consequently, the IC_50_ values of ZnO NPs for both cells were calculated after 24 h of exposure. The calculated IC_50_ value was within the range of 35.04 − 44.86 μg/mL for MCF-7 cells and 55.54 − 63.71 µg/mL for MDA-MB-231 cells. Expectedly, the microscopic images revealed that exposure to the ½ IC_50_ value induced mild toxicity and morphological alternations in both MCF-7 and MDA-MB-231 cells (Fig. 4B). However, when cells were exposed to the IC_50_ value of ZnO NPs, they experienced severe toxicity, leading to a notable decline in their typical adhesion capacity and showing morphological changes, such as the entire collapse of normal spindle shape and cell shrinkage. Similar results were reported for the cytotoxicity observed in cancer cells following treatment with biogenic ZnO NPs. For example, Hussain et al. observed that the plant-mediated biosynthesis of ZnO NPs utilizing the aqueous leaf extract of Pandanus odorifer exhibited the capability to suppress MCF-7, HepG2, and A-549 cancer cell growth at concentrations of 50 and 100 μg/mL^45^. Moreover, Moghaddam et al. found that the IC_50_ value of the biosynthesized ZnO NPs using the Pichia kudriavzevii GY1 yeast strain at 24 h was determined to be 121 µg/mL for MCF-7 cells^12^. Additionally, in the study by Stepankova et al., it was revealed that the as-prepared ZnO NPs exhibited anticancer efficacy against MDA-MB-231, with an IC_50_ value falling within the range of 65–110 µg/mL^13^. Besides, several studies have highlighted the anti-tumor-promoting effect of *R. coriaria* fruit extract against various cancer types^46^. It displayed the ability to significantly decrease the cell viability of the HT-29 and Caco-2 colorectal cancer cells, suppress the proliferation of HT-29 cell colonies, and reduce the growth of mice bearing HT-29 tumor^47^. Another study revealed that it could inhibit the proliferation of MCF-7, T47D, and MDA-MB-231 breast cancer cells at varied times and dose patterns and induce senescence and cell cycle arrest at the G1 phase^48,49^. In the present study, the aqueous extract of *R. coriaria* fruit extract did not induce a significant cytotoxic effect on both cell lines at a concentration of 12.5 μg/mL. However, as the concentration increased, it exhibited moderate cytotoxic activity, leading to a decrease in cell viability percentages to around 66.9% and 70.1% for MCF-7 and MDA-MB-231 cell lines at the concentration of 200 μg/mL. For further investigation of the mechanism of action of ZnO NPs, the MDA-MB-231 cells were selected as a cancer model. The choice of these cells stems from their well-established representation of triple-negative breast cancer characteristics. This type of hard-to-treat cancer represents a significant challenge in cancer research due to its aggressive nature, lack of hormone receptors, and high metastatic potential.

### ZnO NPs induce DNA damage, apoptosis, and S-phase arrest in MDA-MB-231 cells

**Figure 5:**
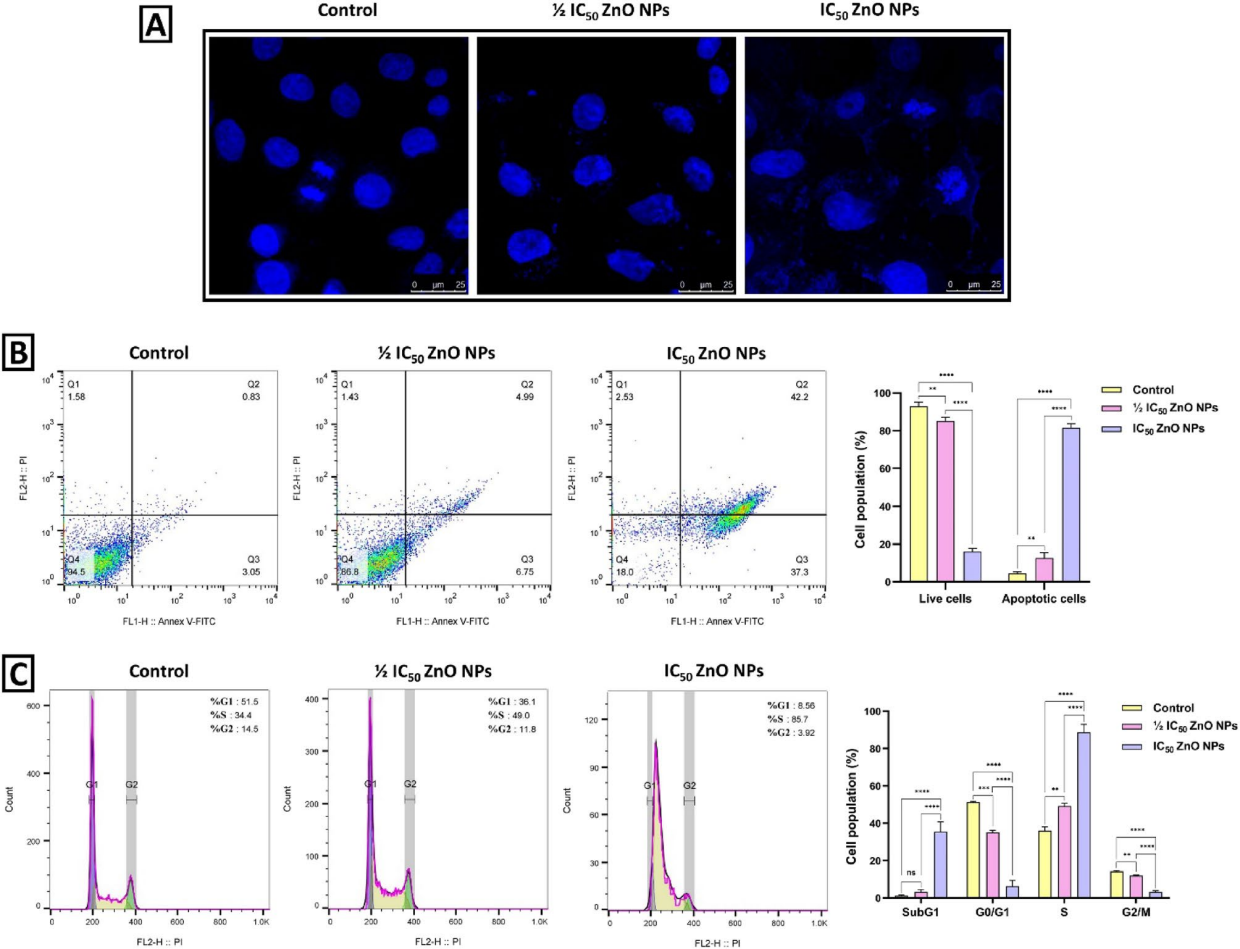
ZnO NPs induce DNA damage, apoptosis, and S-phase arrest in MDA-MB-231 cells. (**A**) Confocal microscopy images of DAPI staining, demonstrating nuclear morphological changes in MDA-MB-231 cells after treatment with ½ IC_50_ and IC_50_ values of ZnO NPs for 24 h. (**B**) Apoptosis analysis was carried out via flow cytometry using Annexin V-FITC/PI staining in MDA-MB-231 cells following exposure to ½ IC_50_ and IC_50_ values of ZnO NPs for 24 h. (**C**) Cell cycle analysis was carried out by flow cytometry using PI staining of MDA-MB-231 after treatment with ½ IC_50_ and IC_50_ values of ZnO NPs for 24 h. All data were presented as mean ± SD and analyzed using one-way ANOVA (Turkey’s test); ****P-value signifies < 0.0001, ***P-value signifies < 0.001, **P-value signifies < 0.01, and *P-value signifies < 0.05.

Apoptosis, a programmed series of events that orchestrates cell death, plays a pivotal role in eliminating cells without causing harm to neighboring cells^12^. It is integral to sustaining the equilibrium between cell proliferation and cell death, which contributes to normal tissue homeostasis. Many NPs have been noted to induce apoptosis in cancer cells, making them potential anticancer agents^45^. Herein, DAPI staining, known for its specific binding to DNA, was used to identify apoptosis-driven nuclear morphological changes in MDA-MB-231 cancer cells after treatment with ½ IC_50_ and IC_50_ values of ZnO NPs for 24 h. As shown in Fig. 5A, the confocal micrograph indicated that the MDA-MB-231 cells exhibited a mild change in the number of apoptotic cells with slightly fragmented nuclei after the exposure to ½ IC_50_ value of ZnO NPs for 24 h, compared to the untreated control cells. Remarkably, when the cells were subjected to the IC_50_ value of ZnO NPs, they exhibited intense DNA damage, coupled with a considerable loss in cell structural integrity and severe signs of apoptosis, including membrane blebbing, cell shrinkage, nucleus fragmentation, and the formation of apoptotic bodies. To validate the apoptosis-mediated activity of ZnO NPs, untreated and treated MDA-MB-231 cells were stained using Annexin V-FITC/PI staining and quantified by flow cytometry. The viable and apoptotic cell populations of MDA-MB-231 cells were analyzed, as illustrated in Fig. 5B. The outcomes confirmed that cells underwent apoptosis following a 24-h treatment with ½ IC_50_ and IC_50_ values of ZnO NPs. In brief, the percentage of live and apoptotic cell populations within the untreated control group averaged 92.83% and 4.44%, respectively. In contrast, when cells were treated with the ½ IC_50_ value, they experienced a drop in viable cell population to 85%, coupled with an increase in apoptotic cell population, averaging 12.59%. As expected, the treated cells with the IC_50_ value showed a significant reduction in viable cell population, reaching 16.03% while the percentage of apoptotic cells increased to 81.57%. These findings agree with several studies showing that ZnO NPs induce apoptosis in various cell lines. For example, 24 h exposure to ZnO NPs resulted in apoptosis in MCF-7 cells, with an early apoptotic cell percentage of 62.53 ± 6.02% for the IC_25_ value, 68.32 ± 2.10% for the IC_50_ value, and 61.85 ± 3.68% for the IC_75_ value^12^. Additionally, an increase in the late apoptotic cell population was observed, which was measured to be 8.51%, 24.47%, and 30.50% for IC_25_, IC_50_, and IC_75_ values, respectively. On the other hand, the viable cell percentages for IC_25_, IC_50_, and IC_75_ values experienced a significant reduction and were found to be 27.94%, 5.98%, and 5.99%, respectively. Another study demonstrated that the eco-friendly ZnO NPs derived from *Annona muricata* induced apoptosis in A549 cells after 24 h of exposure^50^. Flow cytometry analysis demonstrated that following treatment with the ½ IC_50_ value, the percentage of viable cells decreased to 14.05%, while apoptotic cells increased to 84.55%. Furthermore, the percentages of viable cells and apoptotic cells for the IC_50_ value were determined to be 92.55% and 6.66%, respectively.

The cell cycle distribution of the control and treated groups was analyzed by propidium iodide (PI) staining using flow cytometry. It has been established that when cells are exposed to apoptosis-inducing agents, a subset of the cell population emerges before the G1 peak, known as the sub-G1 or apoptosis peak^12^. The diminished stability of this population is attributed to endonuclease activation and subsequent cellular DNA damage. As depicted in Fig. 5C, the results implicated that the exposure of MDA-MB-231 cells to ½ IC_50_ and IC_50_ values of ZnO NPs for 24 h increased the percentage of the sub-G1 population in a dose-dependent manner. Specifically, it was observed that the percentage of the sub-G1 population was 3.33% following ½ IC_50_ value and 35.53% after exposure to IC_50_ concentration, in contrast to the control group, which registered 1.18%. Besides, during the G1 phase, cells enlarge, initiate RNA production, and participate in protein synthesis to support cellular growth and prepare for the subsequent DNA synthesis in the S phase^51^. The analysis showed a significant reduction in G1 phase cells among the treated groups, with a percentage of 35.1% for the ½ IC_50_-treated group and 6.31% for the IC_50_-treated group while the percentage of G1 phase cells in the control group was recorded at 51.3%. On the other hand, ZnO NPs promoted cell cycle arrest in the S-phase, showing a dose-dependent manner to induce DNA damage. Noteworthy, the regulation of the S-phase is governed by the surveillance of replication checkpoints and the control of DNA synthesis^51^. When DNA damage occurs, this checkpoint hinders the progression of the cell cycle. Consequently, the increase in cells in the S-phase could be associated with the incorporation of NPs into impaired DNA during DNA replication. Here, compared to the control group, the treated MDA-MB-231 cells with the ½ IC_50_ value exhibited a high percentage of cells in the S-phase, with a moderate change from 36.07 to 49.23%. Additionally, the cells treated with the IC_50_ value demonstrated a marked elevation in cell percentage in the S-phase, reaching 88.67%. During the G2 phase, cells undergo further growth and replicate their organelles in preparation for cellular division^12^. However, upon treatment with ZnO NPs, a significant reduction was observed in G2 phase cells among the treated groups, with a percentage of 11.97% for the ½ IC_50_-treated group and 3.24% for the IC_50_-treated group while the percentage of G2 phase cells in the control group was recorded at 14.2%.

### ZnO NPs hinder the ability of MDA-MB-231 cells to form colonies and undergo metastasis

**Figure 6:**
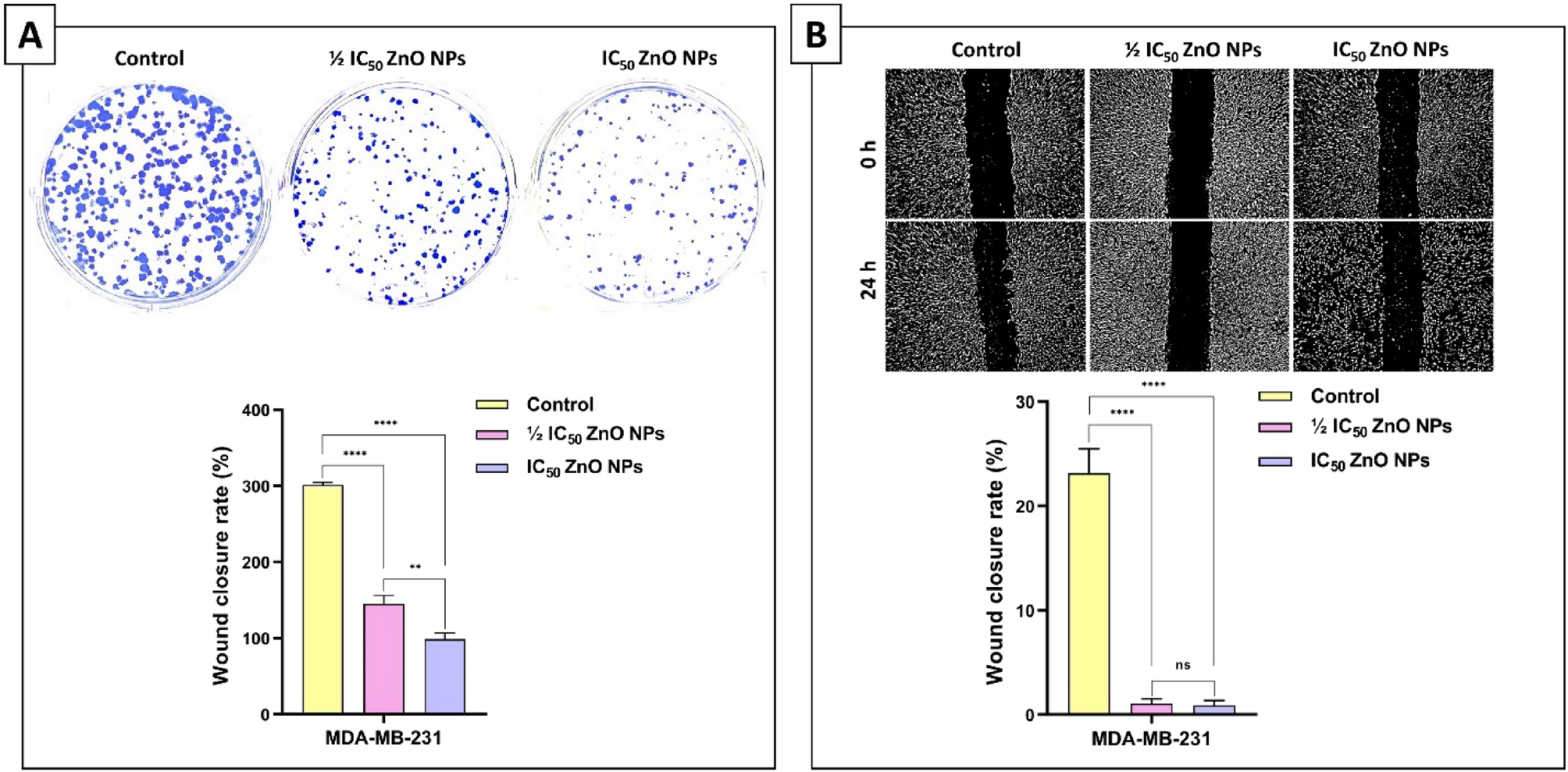
ZnO NPs hinder the ability of MDA-MB-231 cells to form colonies and undergo metastasis. (**A**) In vitro colony formation of MDA-MB-231 cells following treatment with ½ IC_50_ and IC_50_ values of ZnO NPs for 24 h. (**B**) wound healing assay of MDA-MB-231 cells after 24 h of exposure to ½ IC_50_ and IC_50_ values of ZnO NPs. All data were presented as mean ± SD and analyzed using one-way ANOVA (Turkey’s test); ****P-value signifies < 0.0001, ***P-value signifies < 0.001, **P-value signifies < 0.01, and *P-value signifies < 0.05.

In vitro colony formation was performed to evaluate the ability of individual cells to grow into colonies^52^. This study examined the effect of green-synthesized ZnO NPs on colony formation to evaluate each cell's potential for sustained division during population growth. As indicated by Fig. 6A, the ZnO NPs can markedly reduce the colony growth of MDA-MB-231 cells at varying doses, showing statistically significant differences in the number of colonies among the control and treated groups. Briefly, the group subjected to the ½ IC_50_ value showed a noticeable decline in colony formation, with an average count of 145 colonies whereas the control group displayed an average colony count of approximately 301 colonies. However, the group treated with the IC_50_ value displayed a significant decrease in the number of colonies, registering an average count of about 99 colonies. Consequently, the survival fractions of MDA-MB-231 exposed to ½ IC_50_ and IC_50_ were found to be 48.12% and 32.74%, respectively. On the other hand, the wound healing assay has been extensively employed to assess cell migration over a specific duration^53^. Herein, the impact of ZnO NPs on the migration rate of MDA-MB-231 cell lines was assessed after 24 h. As shown in Fig. 6B, the microscopic images of the scratch assay revealed that ZnO NPs notably inhaled cell metastasis. Relative to the control group, it was found that the cells subjected to ½ IC_50_ showed a significant decrease in wound closure rates, measuring at 1.05%, while the control group registered 23.13%. Moreover, the cells subjected to the IC_50_ value displayed a decrease in the wound closure rate of 0.89%. This finding demonstrates that the synthesized ZnO NPs exhibit promising anti-migration characteristics.

### ZnO NPs alter the expression level of genes involved in apoptosis and metastasis

**Figure 7:**
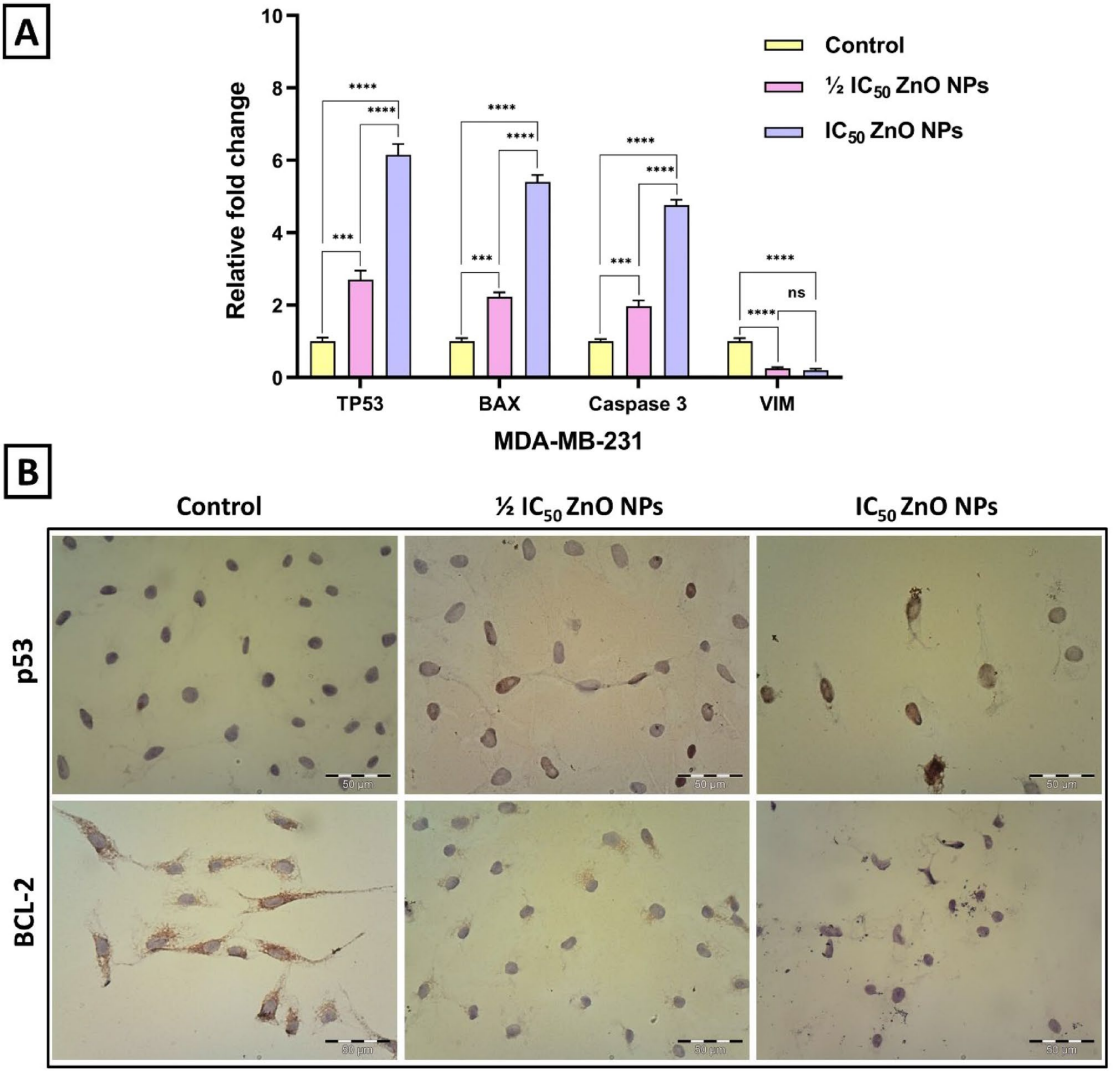
ZnO NPs alter the expression level of genes involved in apoptosis and metastasis. (**A**) The relative fold change in expression levels of TP53, BAX, Caspase 3, and VIM of MDA-MB-231 cells after treatment with ½ IC_50_ and IC_50_ values of ZnO NPs for 24 h. RT-PCR analysis was presented as mean ± SD and analyzed using one-way ANOVA (turkey’s test); ****P-value signifies < 0.0001, ***P-value signifies < 0.001, **P-value signifies < 0.01, and *P-value signifies < 0.05. (**B**) Light microscopic images showed the expression levels of p53 and BCL-2 proteins in MDA-MB-231 cells via immunocytochemical staining after 24 h of treatment with½ IC_50_ and IC_50_ values of ZnO NPs.

Several studies have delved into alternations in breast cancer cell behavior at the molecular level following treatment with ZnO NPs^54^. In this study, RT-PCR was applied to examine the mRNA expression levels of apoptotic markers including TP53, BAX, and Caspase 3, as well as the mesenchymal marker vimentin (VIM) in MDA-MB-231 cells after 24 h treatment with ½ IC_50_ and IC_50_ values of ZnO NPs (Fig. 7A). In the treated MDA-MB-231 cells, the relative mRNA expression levels of the TP53 gene were found to increase by 2.71-fold at the ½ IC_50_ value and 6.15-fold at the IC_50_ value. Moreover, a significant upregulation in BAX expression levels was observed, approximately 2.23-fold and 5.43-fold at the concentrations of ½ IC_50_ and IC_50_, respectively. Notably, Caspase 3 expression levels exhibited elevation by approximately 1.97-fold and 4.7-fold at ½ IC_50_ and IC_50_ concentrations, respectively. On the other hand, VIM expression levels have also been analyzed in MDA-MB-231 cells. It is known that elevated levels of VIM facilitate the attenuation of epithelial characteristics and the acquisition of mesenchymal traits, leading to metastasis^55^. As predicted, ZnO NPs showed anti-metastatic effects on MDA-MB-231 cells. The result indicated a significant reduction in VIM level, showing a marked decline in VIM expression by 0.26-fold at the ½ IC_50_ value and 0.19-fold at the IC_50_ value.

**Figure 8:**
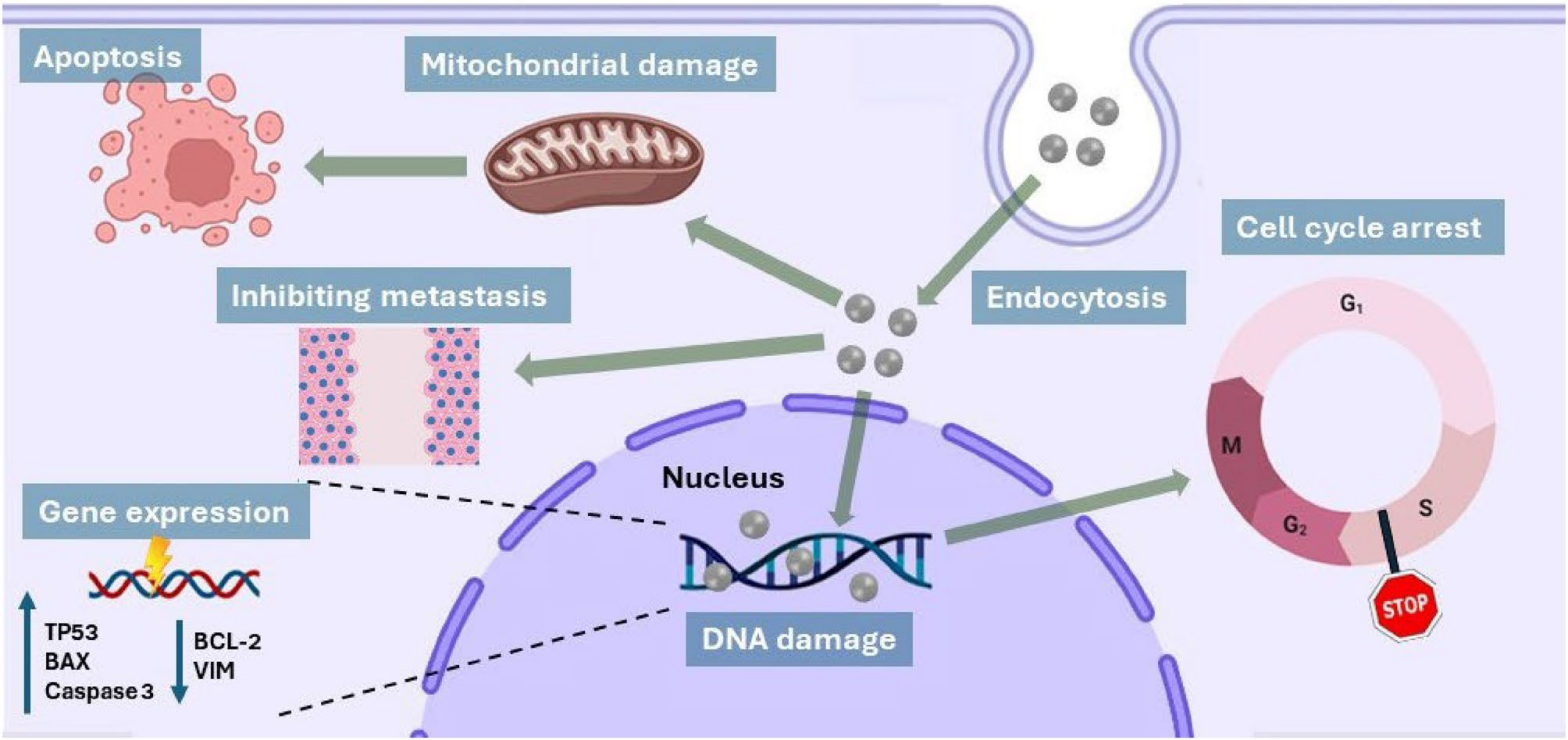
Schematic representation of the possible cytotoxicity mechanisms of ZnO NPs.

Furthermore, immunocytochemical staining was performed to explore the protein expression levels of p53 and Bcl-2 in the untreated control and treated cells. p53 and BCL-2 proteins play a critical role in apoptosis regulation, where p53 is identified as a tumor suppressor protein and BCL-2 acts as an anti-apoptotic protein. The upregulation of p53 expression and the downregulation of Bcl-2 expression induced mitochondrial damage and triggered intrinsically mediated apoptosis^56^. As depicted in Fig. 7B, the outcomes reflected that the expression level of p53 protein has increased in both treated MDA-MB-231 cells in contrast to the control cells. Images revealed that the treated MDA-MB-231 cells with the IC_50_ value possessed a more significant increase in the expression level of p53 protein compared to the group treated with the ½ IC_50_ value. Conversely, the treated cells possessed a notably decreased level of BCL-2 protein when compared to untreated control cells. However, as expected, the expression pattern of BCL-2 was greater at the IC_50_ value than that found in the treated group at the ½ IC_50_ value. This observation aligns with the previous results that ZnO NPs induce apoptosis in MDA-MB-231 cells in a dose-dependent manner. Figure 8 represents a schematic drawing illustrating the possible cytotoxicity mechanisms of the ZnO NPs in this study.

## Conclusion

In this study, *R. coriaria* fruit extract functioned as a reducing and capping agent for synthesizing ZnO NPs through an environmentally friendly and biologically safe approach. The biosynthesized ZnO NPs had an average crystalline size of 16.69 nm and an average grain size of 20.51 ± 3.90 nm. In addition, the elemental composition analysis was studied via EDX, while the presence of phytocompounds on their surface was validated by FT-IR. The stable formation of the biosynthesized NPs was further evidenced by a ζ-potential value of -19.9 ± 0.1663 mV. Moreover, the anti-tumor efficacy of the NPs was evaluated. They exhibited potent anti-cancer effects against MCF-7 and MDA-MB-231 breast cancer cells, with IC_50_ values ranging from 35.04 − 44.86 μg/mL for MCF-7 and 55.54 − 63.71 µg/mL for MDA-MB-231 cells after 24 h of exposure. Mechanistic studies revealed apoptosis induction, with 12.59% and 81.57% apoptosis observed at ½ IC_50_ and IC_50_ values, respectively, in MDA-MB-231 cells. Additionally, ZnO NPs provoked S-phase arrest and inhibited colony-forming and metastatic potential by modulating apoptosis and metastasis-related genes. These findings show the potential of ZnO NPs synthesized from *R. coriaria* fruit extract in cancer treatment and open new avenues for cancer nanotechnology research.

## Materials and methods

### Chemicals and reagents

All chemicals used in this study were of high analytical grade. Zinc acetate dihydrate (Zn (C_2_H_3_O_2_)_2_.2H_2_O, 99.0%), MTT, and Hoechst 33342 were purchased from Sigma-Aldrich, USA. Annexin V-FITC was obtained from BD Pharmingen™, USA. Propidium iodide (PI)/RNAse staining solution was procured from ThermoFisher, USA. Glassware was rinsed in aqua regia, washed with double-distilled water, and oven-dried. All solutions were prepared using double-distilled water. Human breast cancer cell lines MCF-7 and MDA-MB-231 cells were purchased from VACSERA (Dokki, Giza, Egypt). These cells were maintained in Dulbecco's Modified Eagle Medium (DMEM) with high glucose (Capaciron Scientific, Germany), including 10% fetal bovine serum (FBS) (Capaciron Scientific, Germany), at 37 °C in a humidified atmosphere with 5% CO_2_ at 37 °C.

### Preparation of *R. coriaria* fruit aqueous extract

Fresh *R. coriaria* fruits were obtained from a local market in Alexandria, Egypt. The fruits were thoroughly washed with running tap water, followed by double-distilled water to remove any impurities. After overnight drying in the dark at room temperature, the fruits were finely ground using a domestic blender. Then, 7 g of fruit powder was transferred into a flask containing 50 mL of double-distilled water, sealed, and placed on a magnetic stirrer with a speed of 750 rpm at 60 °C for 2 h. Consequently, the resultant extract was filtered repeatedly using Whatman filter paper grade 1, and the purified pale red filtrate was preserved at 4 °C for future use.

### Green synthesis of ZnO NPs

**Figure 9:**
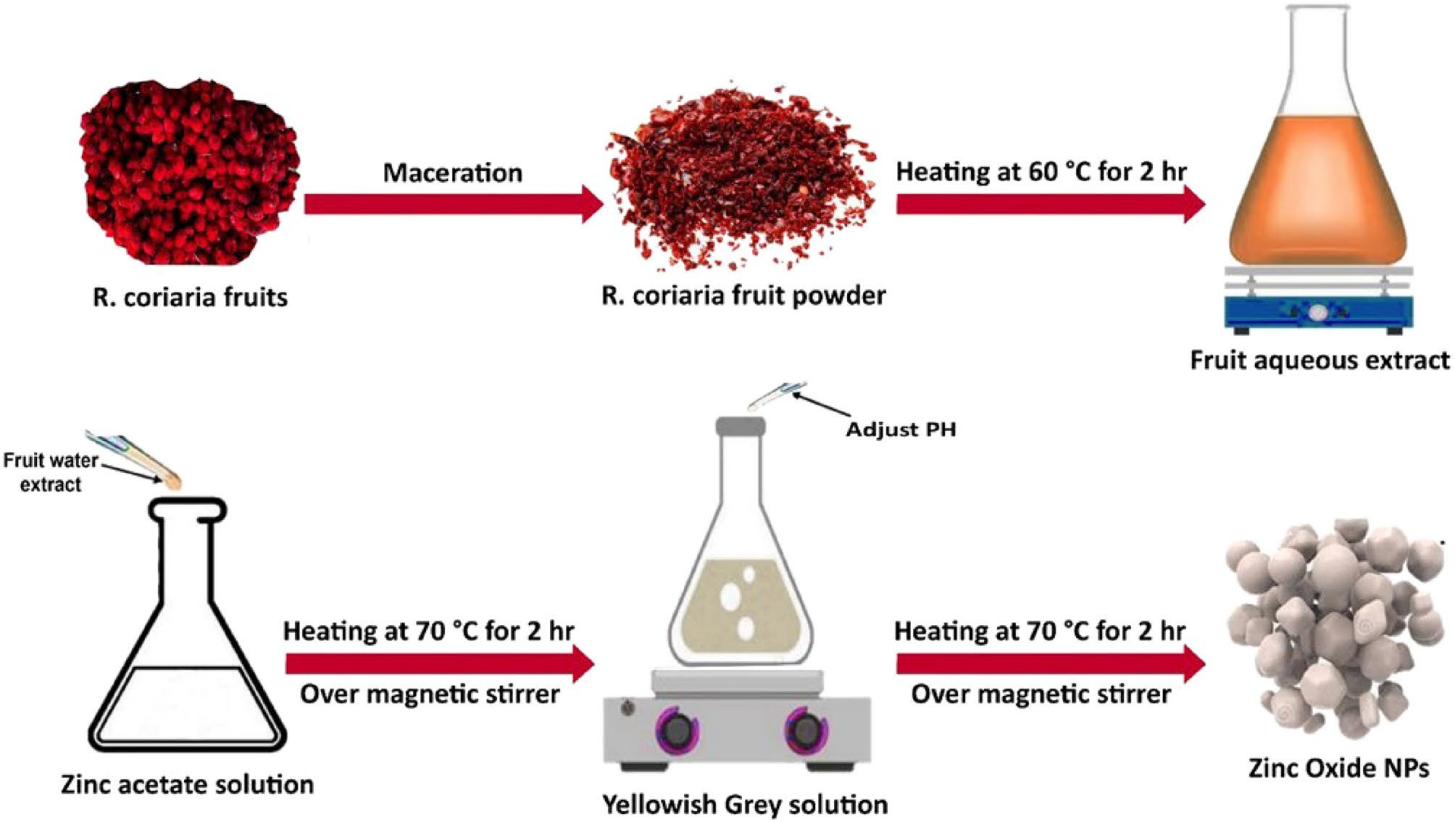
Schematic diagram of the green synthesis of ZnO NPs using *R. coriaria* fruit aqueous extract.

The biosynthesis of ZnO NPs was performed according to previous studies with some modifications^50,57^. In this procedure, 50 mL of an aqueous solution of 0.1 M zinc acetate dihydrate (Zn (C_2_H_3_O_2_)_2_.2H_2_O) was prepared under moderate-speed stirring at room temperature. Then, 25 mL of the extract was added in a dropwise manner into the resultant aqueous solution, and the mixture was then heated at 70 °C for 2 h. As depicted in Fig. 9, the formation of dispersed particles was observed with a notable gradual change in color to yellowish gray. The pH of the reaction was adjusted to 9. Subsequently, the beaker was sealed and placed on a magnetic stirrer with a speed of 400 rpm at 70 °C for 2 h. Afterward, the solution was left to settle and placed into centrifugation at 10000 × g for 10 min. The supernatant was then removed, and the precipitate was washed multiple times with double-distilled water, followed by ethanol, and left to dry in an oven at 80 °C overnight. Eventually, the dried powder was ground using an agate mortar and pestle and transferred into a glass vial.

### Physicochemical characterization of ZnO NPs

#### Optical properties

The as-prepared NPs were dispersed in deionized water, sonicated for 10 min, and extensively characterized. The optical characteristics of NPs were analyzed by measuring the absorption maxima in the wavelength range of 200–600 nm at room temperature using a double-beam UV–Vis spectrophotometer (Shimadzu UV-1800, Japan)^58^. The SPR of electrons on the metal oxide NP surface displays distinctive peaks at specific wavelengths.

#### Structural properties

The crystalline nature, purity, and size of ZnO NPs were analyzed by XRD (Siemens D-5000). The XRD diffraction pattern was observed using Cu Kα radiation (λ = 1.54184 Å) employing a voltage of 30 kV, an electric current of 30 mA, and scanning within the 2θ angular range of 2°–100°. Furthermore, the average crystallite size of ZnO NPs was estimated using the Debye–Scherrer Eq. ^59^:

$$D = 0.9\frac{{{\text{K}}\lambda }}{{\beta \cos {\uptheta }}}$$

where (D) is the average crystallite size, k is the Scherrer's constant (0.9), λ is the wavelength of 1.5406 Å, β is the full width at half maximum of the specified diffraction peak associated with the (101) plane, and θ is the Bragg angle.

#### Morphological properties

The particle size and morphology of the biosynthesized NPs were investigated by TEM (JEOL JEM-1400 Plus), operated at 80 kV ^60^. The micrograph, captured at a magnification of 100 nm, was used to calculate the average particle size with the assistance of Image J software.

#### Compositional properties

The elemental composition of ZnO NPs was examined using SEM (JEOL JSM-IT200) coupled with an energy-dispersive X-ray instrument. The procedure involved placing ZnO NPs onto the stub using carbon tape, followed by fixation and gold coating via sputtering. Subsequently, the prepared stub was introduced into the instrument chamber for analysis^58^.

#### Functional properties

The vibration modes of the active functional groups onto the surface of biogenetic ZnO NPs, which engaged in reducing and stabilizing the NPs, were analyzed using FT-IR (Bruker FT-IR Spectrometer). The FT-IR spectra of *R. coriaria* fruit aqueous extract and as-prepared NPs were scanned using the potassium bromide (KBr) disk method over the range of 4000–450 cm^−1^^58^.

#### Surface charge properties

To identify the magnitude of the electrical charge at the particle surface, the ζ-potential of biosynthesized NPs was measured by Zetasizer (Malvern Zetasizer Ultra, UK) in water as a dispersant^58^. The samples of ZnO NPs were diluted ten times using double-distilled water and then transferred to a DTS1070 cell at 25 °C. The data was analyzed using ZS XPLORER v.1.3.2 software.

## in vitro anticancer studies

### MTT assay

MCF-7 or MDA-MB-231 cell lines were seeded in a 96-well plate (5000 cells/well) for 24 h. The attachment cells were subjected to ZnO NPs and *R. coriaria* fruit aqueous extract at serial concentrations, ranging from 12.5 to 200 µg/mL, for 24 h. To evaluate the cytotoxicity, the medium in each well was replaced with 100 µl of (0.5 mg/mL) MTT solvent. After 4 h of incubation in the dark at 37 °C, the medium was carefully removed, and 100 μL of DMSO was introduced to every well and properly mixed for an additional 10 min. The absorbance was measured at a wavelength of 570 nm using a microplate reader (Bio-Rad, CA, USA). The cell viability was determined using the following formula:

$$Cell \,viability \left( \% \right) = \frac{OD\, value\, of\, samples}{{OD\, value\, of\, Controls}} \times 100$$

The half-maximal inhibitory concentration (IC_50_) values were conducted using the GraphPad Prism version 9 software. Additionally, the cellular morphology of untreated and treated cells was visualized using a phase-contrast inverted microscope with a digital camera (Olympus, Japan).

### Evaluation of DNA damage via confocal microscopy

To examine the DNA damage triggered by ZnO NPs, MDA-MB-231 cells were cultured at a density of 1 × 10^5^ cells on glass coverslips in a 12-well plate for 24 h. Following this, cells were exposed to ½ IC_50_ and IC_50_ values of ZnO NPs for 24 h. Then, untreated and treated cells were washed with phosphate-buffered saline (PBS) (Capaciron Scientific, Germany) and fixed with 4% paraformaldehyde for 30 min. To promote permeabilization, 0.5% Triton X-100 was applied to cells for 10 min, followed by washing three times with PBS. DAPI staining (1 μg/mL) was applied to cells for 5 min at room temperature. Images were captured using a Leica laser scanning confocal microscope (Leica DMi8, Wetzlar, Germany).

### Flow cytometry analysis of apoptotic cells

The apoptosis-driven anticancer properties of ZnO NPs were measured by the quantification of the annexin-positive cells as an indicator of apoptosis using an Annexin V-fluorescein isothiocyanate (FITC)/PI staining kit. Briefly, MDA-MB-231 Cells were seeded (1 × 10^5^ cells/well) in a 6-well plate for 24 h, followed by treatment with ½ IC_50_ and IC_50_ values of ZnO NPs. After 24 h, untreated and treated cells were harvested, washed twice with PBS, and resuspended in PBS. Subsequently, the Annexin V-FITC/PI staining kit was incubated with the cells according to the manufacturer’s protocol. The gating of viable cells and apoptotic cells was carried out using FlowJo v10.8 software.

### Cell cycle analysis by flow cytometry

To access the cell cycle distribution of MDA-MB-231 cells, 1 × 10^5^ cells per well were plated in a six-well plate. Then, the cells were exposed to ½ IC_50_ and IC_50_ values of ZnO NPs for 24 h. Following trypsinization, cells were PBS-washed, fixed in 70% pre-cooled ethanol, and stored at − 20 °C. Subsequently, after resuspension in 1 × PBS (pH 7.4), cells were treated with 50 μg/mL PI/RNAse solution for 30 min in the dark at room temperature. Then, the cell cycle analysis was performed and analyzed using FlowJo v10.8 software.

### Colony formation assay

For the clonogenic assay, MDA-MB-231 cells were seeded in a 6-well plate (400 cells/well) for 24 h. Afterward, the cells were subjected to ½ IC_50_ and IC_50_ values of ZnO NPs for 24 h and then cultured for approximately 10 days. Consequently, the colonies were washed with PBS and fixed with 4% paraformaldehyde for 15 min, followed by staining with 1% crystal violet for 30 min. Colonies of untreated and treated cells were counted and photographed.

### Analysis of cell migration using wound-healing assay

MDA-MB-231 cells were cultured (1 × 10^5^ cells/well) in a 12-well plate for 24 h in a complete culture medium. After reaching 95–100% confluence, a scratch was made using a P200 pipette tip, followed by double washes with PBS to remove any cell debris. Subsequently, cells were treated with ½ IC_50_ and IC_50_ values of ZnO NPs for 24 h. Later, the wound areas of untreated and treated cells were photographed under the microscope and measured using ImageJ software at two different time points (0 and 24 h).

### Real-time PCR analysis for TP53, BAX, Caspase 3, and Vimentin expression

The total RNAs of treated and untreated MDA-MB-231 cells were isolated using the RNeasy Mini kit (Qiagen, Germany), according to the provided manufacturer's instructions. The purity and concentration of the extracted RNAs were measured using a Nanodrop 2000 spectrophotometer (Thermo Fischer Scientific, USA). Then, cDNA was synthesized using the TOPscript™ cDNA Synthesis Kit (Enzynomics, South Korea). The real-time PCR was conducted by a real-time PCR system (Rotor-Gene Q, Qiagen) using SYBR Green qPCR Premix (Enzynomics, South Korea). GAPDH was utilized as a reference gene, and the relative expression levels were calculated using the 2^−△△Ct^ method. The primer sequences (forward and reverse) were: 5′-CCTCAGCATCTTATCCGAGTGG-3′, and 5′-TGGATGGTGGTACAGTCAGAGC-3′ for the TP53 gene; 5′-CTCTGAGCAGATCATGAAGACAGG-3′, and 5′-GAAAACATGTCAGCTGCCACTCG-3′ for the BAX gene; 5′-TCTGGTTTTCGGTGTGTGTG-3′, and 5′-CGCTTCCATGTATGATCTTTGGTT-3′ for the Caspase 3 gene; 5′-TCCAAGTTTGCTGACCTCTCTG-3′, and 5′-CAGTGGACTCCTGCTTTGCC-3′ for the Vimentin gene; and 5′-CCAAAATCAAGTGGGGCGAT-3′, and 5′-GGCAGAGATGATGACCCTTT-3′ for the GAPDH gene.

### Immunocytochemical analysis of p53 and BCL-2 expression

Immunocytochemical staining was performed using the Autostainer Link 48 immunostaining platform (Agilent Technologies) for both untreated cells and treated MDA-MB-231 cells. The antibody sources and dilutions included p53 (DAKO, Agilent, clone DO-7, prediluted) and BCL-2 (DAKO, Agilent, clone 124, prediluted). After fixation, the antigenic retrieval was conducted using EnVision FLEX Target Retrieval High pH Solution (Dako Omnis) at 99 °C for 30 min. The endogenous peroxidase activity was inhibited using the EnVision Flex Peroxidase-blocking reagent (Dako Omnis) for 5 min. Subsequently, the ready-to-use p53 or BCL-2 primary antibodies were incubated for 30 min or 20 min. The signal amplification was performed using EnVision™ Flex HRP (Dako Omnis) for 15 min, followed by signal detection for 10 min using EnVision™ FLEX DAB + Chromogen (Dako Omnis). Additionally, counterstaining was carried out using EnVision Flex hematoxylin (Dako Omnis) for 10 min. The stained samples were examined under a light microscope, and images were captured.

### Statistical analysis

The statistical analysis was performed using GraphPad Prism version 9 software. At least three separate repeats were interpreted as the mean ± standard deviation (SD). Data analysis was performed using the one-way ANOVA (Turkey’s test) to detect differences between groups, where differences were considered statistically significant at *P* < 0.05.

## Data Availability

All data generated or analyzed during the current study are available from the corresponding author upon reasonable request.
